# Economic Burden of HIV in a Commercially Insured Population in the United States

**DOI:** 10.36469/001c.56928

**Published:** 2023-01-19

**Authors:** Cindy Y. Chen, Prina Donga, Alicia K. Campbell, Babafemi Taiwo

**Affiliations:** 1 Janssen Scientific Affairs, LLC, Titusville, New Jersey; 2 Division of Infectious Diseases, Northwestern University Feinberg School of Medicine, Chicago, Illinois

**Keywords:** human immunodeficiency virus, economic burden, healthcare resource utilization, healthcare costs, pre-exposure prophylaxis, newly diagnosed HIV

## Abstract

**Background:** With advances in antiretroviral therapy (ART), people with HIV infection are living longer. Pre-exposure prophylaxis (PrEP) to reduce HIV infection risk continues to be underutilized in high-risk individuals. Recent data on economic burden for patients with newly diagnosed HIV-1 or initiated with PrEP are limited.

**Objectives:** To assess characteristics, healthcare resource utilization (HRU), and costs among adults and adolescents either with newly diagnosed HIV-1 or initiated with PrEP.

**Methods:** This retrospective observational study utilized data from the IBM MarketScan® Commercial Claims and Encounters database. Adults with newly diagnosed HIV-1 or those initiated with PrEP were included (index date was the first HIV diagnosis or PrEP prescription, respectively, between January 1, 2016, and April 30, 2021). Corresponding cohorts of adolescents were considered exploratory. Descriptive analyses were conducted to assess baseline demographics and clinical characteristics, and all-cause and HIV-related HRU and costs per patient per month (PPPM) during follow-up.

**Results:** Data from 18 154 adults and 220 adolescents with newly diagnosed HIV and 34 123 adults and 175 adolescents initiated with PrEP were included. Approximately 70% of adolescents and 9% of adults receiving PrEP were female. Baseline depression/anxiety was present in 16.1% and 24.6% of adults and 14.5% and 45.1% of adolescents in the HIV and PrEP cohorts, respectively. Substance abuse in the HIV and PrEP cohorts, respectively, was reported in 10.1% and 7.0% of adults, and 2.7% and 17.7% of adolescents. During follow-up, among adults with newly diagnosed HIV, mean (SD) total all-cause and HIV-related PPPM costs were 2657(5954) and 1497(4463), respectively; pharmacy costs represented 47% of all-cause costs and 67% of HIV-related costs, but only 37% of patients had an HIV-related prescription. All-cause costs PPPM for adults with PrEP were 1761(1938), with pharmacy costs accounting for 71%.

**Conclusions:** Despite advances in ART, patients with newly diagnosed HIV and at-risk patients receiving PrEP continue to incur HRU costs. The chronic nature of HIV warrants further exploration of factors contributing to disease burden and opportunities to improve prevention strategies.

## BACKGROUND

Human immunodeficiency virus is a chronic infectious disease characterized by a decline in the number of CD4+ T cells, which underlies the immunosuppression observed in affected individuals. At the end of 2019, an estimated 1.2 million people aged 13 years and older in the United States had HIV, including an estimated 159 000 (13%) people whose infections had not been diagnosed; over 15 000 people with HIV died in 2019.^1^ Since the advent of antiretroviral therapy (ART), the infection rate and number of deaths attributed to HIV/AIDS have declined, thus transforming HIV infection from an acute infection with high mortality to a chronic, manageable disease. Still, in 2019, almost 35 000 patients 13 years and older had a new diagnosis of HIV in the United States.^2^ People living with HIV (PLWH) are living longer,^3^ and as they age, they are often at greater risk for poor health outcomes than the general population due to development of additional chronic comorbidities, such as type 2 diabetes, hypertension, and depression.^4–6^ Additional data on the burden of HIV-1 and associated comorbidities in newly diagnosed PLWH will be helpful in contextualizing the cost of illness across the continuum of disease compared with other chronic conditions.

In a recent study of Medicaid-insured PLWH less than 65 years of age, disproportionate numbers of comorbidities and concomitant medications were observed, with greater occurrence of these factors associated with increasing age.^7^ A similar real-world study of those with Medicare Supplemental insurance showed that older PLWH (≥65 years of age) had significantly more non-HIV conditions and non-ART medications than older HIV-negative individuals.^8^ The increased burden of comorbidities and concomitant medication use is also likely to contribute to greater healthcare utilization costs. Indeed, a recent study using claims data from the Veterans Affairs Administration found that PLWH (mean age, 49 years) had greater pharmacy, outpatient, and total healthcare costs than matched controls without HIV.^9^ Similarly, a US HIV Research Network study of healthcare resource utilization (HRU) among adolescents and young adults (aged 13-30 years) with HIV found that increasing age was associated with more emergency department (ED) visits and inpatient days.^10^ Recent studies have shown that mean annual medical costs for PLWH are approximately $30 000 per patient.^11,12^ Moreover, the lifetime HIV-related healthcare cost for PLWH ranged from approximately $350 000 to $620 000 in 2004 US dollars.^13^ A modeling analysis based on a targeted literature review, which considered multiple cost variables inflated to 2017 US dollars, estimated total lifetime costs up to $1.7 million per patient; over time, as predicted survival rates approached those of the general population, costs associated with HIV treatment and adverse events, as well as cost of certain comorbidities increased, while other HIV management costs (eg, outpatient, inpatient day, and ED care, HIV testing, non-HIV medication, and prophylaxis for opportunistic infection) decreased.^14^ The findings described above substantiate a continued unmet need for better prevention strategies and disease management, with the goal of improving outcomes and reducing costs.

HIV pre-exposure prophylaxis (PrEP) has been shown to decrease the risk of HIV infection and therefore has the potential to reduce the incidence of HIV infections in the United States.^15,16^ Two currently available once-daily oral products, emtricitabine 200 mg/tenofovir disoproxil fumarate 300 mg and emtricitabine 200 mg/tenofovir alafenamide 25 mg, were approved by the US Food and Drug Administration (FDA) for PrEP in 2012 and 2019, respectively. A long-acting injectable product, cabotegravir, was approved for PrEP in December 2021.^17^ Previously, a retrospective analysis of US commercial insurance claims data from 2009 to 2014 found an increasing trend in prescriptions for PrEP; however, these are older data and warrant update.^18^ Recent CDC reports estimate PrEP utilization among high-risk individuals for whom PrEP is indicated at 23% in 2019.^1^ Annual costs for patients using HIV PrEP can be substantial, although estimates vary widely, perhaps in part because these patients are more likely than PLWH to have public healthcare coverage or use a publicly sponsored assistance program to pay for medications.^19^

When considering the potential lifetime impact of HIV in those who are at risk or infected, the existing data point to a trend toward increases in comorbidities, HRU, and associated costs among PLWH.^14^ This is likely to increase as patients live longer, given advances in ART. While most affected patients are adults, the populations of interest for this study also include adolescents, who may potentially differ from their adult counterparts in terms of demographics, clinical characteristics, and HRU. Despite the substantial public health, social, and economic implications of this observed trend, recent data on economic burden of HIV or HIV PrEP in a commercially insured population in the United States is limited. This study aims to describe the demographic and clinical characteristics of adults with newly diagnosed HIV-1 or those who initiated PrEP and to assess all-cause HRU (eg, number of admissions, visits, encounters, and prescriptions) and costs, as well as HIV-related HRU and costs in this population. Additionally, we aim to describe the same outcomes in adolescents with newly diagnosed HIV-1 and those who initiated PrEP; these were considered exploratory analyses due to an anticipated small sample size of adolescents in the database.

## METHODS

### Study Design

This retrospective, observational cohort study included adult (aged 18-64 years) and adolescent (aged 10-17 years) patients with newly diagnosed HIV-1 and adult and adolescent patients who initiated HIV PrEP in the United States. Patients were identified from administrative medical and pharmacy claims data from the IBM MarketScan® Commercial Claims and Encounters (CCAE) database from January 1, 2016, through April 30, 2021.

### Data Source

This study utilized administrative medical and pharmacy claims data obtained from the IBM MarketScan® CCAE database, which captures data from 1996 forward, and contains fully adjudicated pharmacy and medical claims of employees and their dependents. It provides information about resource utilization and associated costs for healthcare services performed in both inpatient and outpatient settings for individuals covered annually by a geographically diverse group of self-insured employers and private insurance plans across the United States. Data records were de-identified and certified to be fully compliant with HIPAA (Health Insurance Portability and Accountability Act) patient confidentiality requirements. Institutional review board approval to conduct this study was not required because the study uses only de-identified patient records and does not involve the collection, use, or transmittal of individually identifiable data.

### Selection of Patients With Newly Diagnosed HIV-1

**Figure 1.**
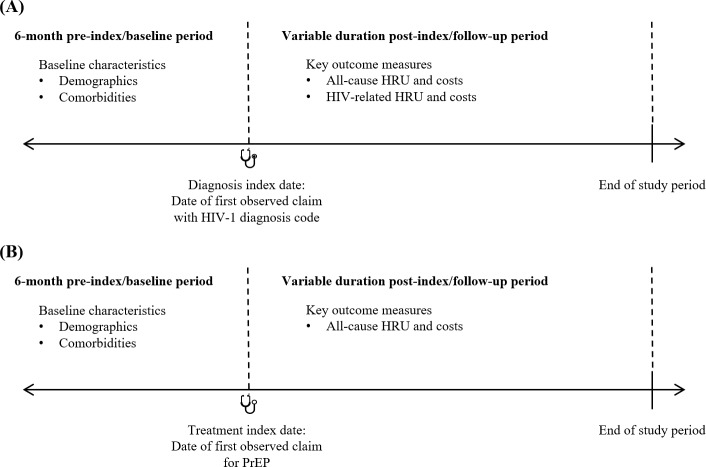
Schematic of Study Design (**A**) Patients with newly diagnosed HIV-1; (**B**) patients initiated with PrEP. Abbreviations: HRU, healthcare resource utilization; PrEP, pre-exposure prophylaxis.

Patients with at least 1 medical claim with an HIV-1 diagnosis code (ICD-9-CM codes 042, 795.71, V08; ICD-10-CM codes B20, R75, Z21; **Supplemental Table S1**) in any diagnosis position between January 1, 2016, and April 30, 2021, were identified as having newly diagnosed HIV-1. The date of the first observed medical claim with HIV-1 diagnosis was defined as the diagnosis index date. Patients were required to have at least 6 months of continuous enrollment in the database prior to and including the diagnosis index date, and the 6-month period prior to the diagnosis index date was defined as the baseline period **(Figure 1A)**. Patients were excluded from the newly diagnosed group if they had at least 1 medical claim with an HIV-1 or HIV-2 diagnosis code **(Supplemental Table S1)** or at least 1 claim for ART **(Supplemental Table S2)** any time prior to the diagnosis index date.

### Selection of Patients Initiated With PrEP

Patients with at least 1 pharmacy claim for PrEP (GPI codes 1210990230%, 12109902290320; **Supplemental Table S1)** between January 1, 2016, and April 30, 2021, were identified. The treatment index date was the date of the first observed pharmacy claim for PrEP. Patients were included if they had at least 6 months of continuous enrollment in the database prior to the treatment index date **(Figure 1B).** Patients were excluded from the PrEP group if they had at least 1 medical claim with an HIV-1, HIV-2, or hepatitis B diagnosis code **(Supplemental Table S1)** or at least 1 claim for hepatitis B–related medications **(Supplemental Table S3)** any time prior to the treatment index date to 30 days after the treatment index date. Patients were also excluded if they had at least 1 claim for ART, including those indicated for PrEP, any time prior to the treatment index date **(Supplemental Table S2)** or if the PrEP prescription claim on the treatment index date had less than a 30-day supply of medication. The latter 2 exclusion criteria were applied to ensure that included patients were naïve to PrEP and to exclude those who may have used the treatment as postexposure prophylaxis.

### Study Cohorts

Both the newly diagnosed HIV-1 group and the PrEP group were stratified by age at their respective diagnosis or treatment index date. The subgroups of adults (18-64 years old) and adolescents (10-17 years old) comprised the 4 cohorts analyzed in this study: adults with newly diagnosed HIV-1, adults initiated with PrEP, adolescents with newly diagnosed HIV-1, and adolescents initiated with PrEP.

### Data Analysis

To describe baseline demographic and clinical characteristics among adults and adolescents with newly diagnosed HIV-1 or initiated with PrEP, means ± SD were reported for continuous variables, and frequencies and proportions were reported for categorical variables. Means ± SD and medians were reported for all-cause and HIV-related costs incurred among adults and adolescents with newly diagnosed HIV-1, and for all-cause costs incurred among adults and adolescents initiated with PrEP. Frequencies and proportions were reported for all-cause and HIV-related HRU, including inpatient admission, ED visits, outpatient and physician office visits, and prescription fills, among adults and adolescents with newly diagnosed HIV-1, and for all-cause HRU among adults and adolescents initiated with PrEP. All data analyses were conducted using SAS Enterprise Guide 7 (SAS Institute, Cary, North Carolina).

### Variables

All analyses were purely descriptive, and no associations or comparisons were made among variables or cohorts. Analyses for the PrEP cohorts were conducted in aggregate and were not stratified by specific medication.

Baseline patient demographics and clinical characteristics were reported for the 4 cohorts. Demographics measured as of the diagnosis or treatment index date included age in years, gender (female/male), US geographic region (Northeast, North Central, South, West, unknown), insurance plan type (health maintenance organization, preferred provider organization [PPO], consumer-directed health plan, other/unknown), and calendar year in which the diagnosis or treatment index date occurred. Clinical characteristics assessed during the baseline period included the Quan-Charlson Comorbidity Index (QCI) score,^20,21^ individual comorbid conditions **(Supplemental Table S4)**, and concomitant medications **(Supplemental Table S5**). The comorbidities assessed were prediabetes, type 2 diabetes, myocardial infarction, peripheral vascular disease, congestive heart failure, hypertension, hyperlipidemia, obesity, nonalcoholic steatohepatitis, cancer, lipodystrophy, opportunistic infections, depression and anxiety, and substance abuse disorders. Concomitant medications of interest were diabetes therapies, psychiatric/neurologic therapies, chronic oral corticosteroids (defined as continuous use of corticosteroids at any dose for ≥60 days, ie, having ≥2 consecutive written prescriptions for steroid with total days supply ≥60 days during the baseline period), hormone therapy/contraception, appetite stimulants/suppressants, statins, and antihypertensives.

Outcomes were assessed during the variable follow-up period after the diagnosis or treatment index date. For the cohorts of adults and adolescents with newly diagnosed HIV-1, all study outcomes were measured from the diagnosis index date to the earlier of end of continuous enrollment in the database or end of the study period. For the cohorts of adults and adolescents initiated with PrEP, all study outcomes were measured from the treatment index date to the earliest of HIV-1 diagnosis **(Supplemental Table S1)**, end of continuous enrollment, or end of the study period. Healthcare resource utilization included inpatient hospitalizations, ED visits, physician office visits, other outpatient service encounters, and prescription fills. Healthcare costs included pharmacy and medical costs; medical costs were stratified by inpatient costs, ED costs, physician office visit costs, and other outpatient costs. All-cause HRU was defined as the percentage of patients who used resources associated with any condition. HIV-related HRU was defined as the percentage of patients who used resources associated with HIV. All-cause total costs were defined as the sum of direct healthcare costs incurred from all medical and pharmacy claims associated with any condition. HIV-related total costs were defined as the sum of all direct healthcare costs incurred from medical claims associated with a diagnosis code for HIV **(Supplemental Table S1)** (specifically, claims for inpatient hospitalizations with a diagnosis code for HIV in the primary diagnosis position or any other medical claims with a diagnosis code for HIV in any diagnosis position) and from pharmacy claims for ART **(Supplemental Table S2)**. The number and percentage of patients utilizing all-cause or HIV-related medical or pharmacy resources were reported. Because a specified minimum duration of continuous enrollment following the index date was not required, HRU and costs (in US dollars) were reported per patient per month (PPPM) to adjust for the variable length of follow-up. All dollar estimates were inflated to 2020 US dollars using the Medical Care Component of the Consumer Price Index.^22^ Outcomes were assessed separately for the cohorts of adults with newly diagnosed HIV-1, adults initiated with PrEP, adolescents with newly diagnosed HIV-1, and adolescents who initiated PrEP.

## RESULTS

**Figure 2.**
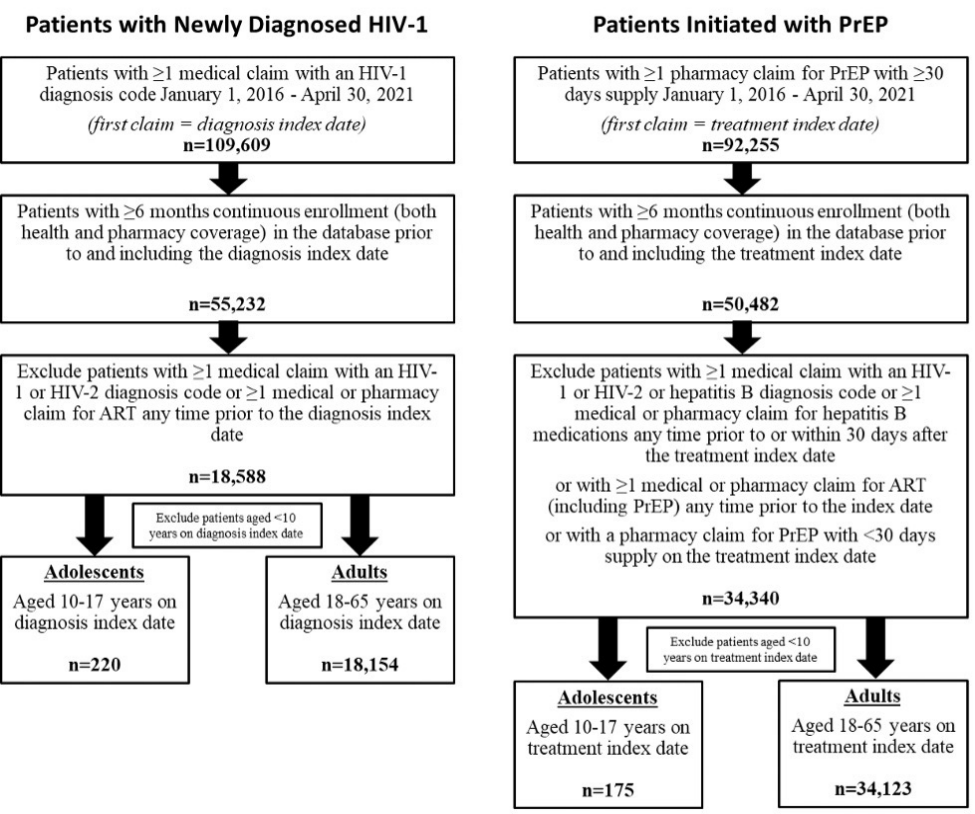
Patient Attrition Abbreviations: ART, antiretroviral therapy; PrEP, pre-exposure prophylaxis.

Between January 1, 2016, and April 30, 2021, a total of 109 609 patients in the dataset had at least 1 medical claim with an HIV-1 diagnosis code, and 92 255 patients had at least 1 pharmacy claim for PrEP with at least 30 days’ supply. Of those patients, only 55 232 (50.4%) and 50 482 (54.7%), respectively, had at least 6 months of prior continuous enrollment documented in the database. After the remaining inclusion and exclusion criteria were applied, a total of 18 588 patients of all ages were identified as newly diagnosed HIV-1 patients, including 220 adolescents and 18 154 adults. The group of patients initiated with PrEP comprised 34 340 patients, including 175 adolescents and 34 123 adults **(Figure 2)**. The IBM MarketScan® CCAE database does not capture data on patients over the age of 64 years; thus, no patients older than 64 years were included in the analysis. A relatively small number of patients aged less than 10 years were identified and were not included.

### Newly Diagnosed HIV-1: Adults

In the cohort of adults with newly diagnosed HIV-1, the mean (SD) age was 38.8 (12.8) years and 63.8% were male. Most patients were from the South (51.1%), followed by the Northeast (25.5%). The most common commercial plan type was PPO (49.7%) **(Table 1).** During the baseline period, mean (SD) QCI score was 0.4 (1.1), and comorbidities that were reported in more than 10% of the cohort included hypertension, hyperlipidemia, depression/anxiety, obesity, and substance abuse disorders **(Table 1).**

**Table 1.** Patient Baseline Demographics and Clinical Characteristics

	**Age Group**			
	**Patients With Newly Diagnosed HIV-⁠1**		**Patients Initiated With PrEP**	
	**10-17 y**	**18-64 y**	**10-17 y**	**18-64 y**
Total No. of patients	220	18 154	175	34 123
Age (y), mean (SD)	14.7 (2.2)	38.8 (12.8)	15.7 (1.4)	34.7 (11.0)
Gender, n (%)				
Male	132 (60.0)	11 578 (63.8)	53 (30.3)	31 037 (91.0)
Female	88 (40.0)	6576 (36.2)	122 (69.7)	3086 (9.0)
Region, n (%)				
Northeast	49 (22.3)	4621 (25.5)	48 (27.4)	7476 (21.9)
North Central	29 (13.2)	2279 (12.6)	50 (28.6)	5398 (15.8)
South	124 (56.4)	9281 (51.1)	59 (33.7)	13 995 (41.0)
West	17 (7.7)	1814 (10.0)	16 (9.1)	7001 (20.5)
Unknown	1 (0.5)	159 (0.9)	2 (1.1)	253 (0.7)
Commercial plan type, n (%)				
HMO	26 (11.8)	1790 (9.9)	28 (16.0)	4770 (14.0)
PPO	104 (47.3)	9019 (49.7)	80 (45.7)	16 363 (48.0)
POS	27 (12.3)	2289 (12.6)	0 (0.0)	3996 (11.7)
CDHP/HDHP	53 (24.1)	3765 (20.7)	39 (22.3)	7235 (21.2)
Others^a^	2 (0.9)	759 (4.2)	0 (0.0)	1011 (3.0)
Unknown	8 (3.6)	532 (2.9)	1 (0.6)	748 (2.2)
Year of diagnosis (newly diagnosed HIV-1) or treatment index date (initiated with PrEP), n (%)				
2016	69 (31.4)	4315 (23.8)	25 (14.3)	5504 (16.1)
2017	43 (19.5)	3876 (21.4)	31 (17.7)	6392 (18.7)
2018	42 (19.1)	3926 (21.6)	40 (22.9)	8123 (23.8)
2019	36 (16.4)	3022 (16.6)	44 (25.1)	7108 (20.8)
2020	23 (10.5)	2441 (13.4)	32 (18.3)	5457 (16.0)
2021^b^	7 (3.2)	574 (3.2)	3 (1.7)	1539 (4.5)
QCI, mean (SD)	0.2 (0.5)	0.4 (1.1)	0.2 (0.4)	0.2 (0.5)
Comorbid condition, n (%)				
Prediabetes	1 (0.5)	187 (1.0)	2 (1.1)	310 (0.9)
Type 2 diabetes	2 (0.9)	1671 (9.2)	0 (0.0)	1454 (4.3)
Cardiovascular disease	1 (0.5)	568 (3.1)	0 (0.0)	302 (0.9)
Hypertension	4 (1.8)	3876 (21.4)	2 (1.1)	4735 (13.9)
Hyperlipidemia	12 (5.5)	3266 (18.0)	4 (2.3)	4920 (14.4)
Obesity	7 (3.2)	2213 (12.2)	11 (6.3)	3199 (9.4)
Nonalcoholic steatohepatitis	0 (0.0)	375 (2.1)	0 (0.0)	441 (1.3)
Cancer	3 (1.4)	635 (3.5)	1 (0.6)	311 (0.9)
Lipodystrophy	0 (0.0)	3 (0.0)	0 (0.0)	5 (0.0)
Opportunistic infections	0 (0.0)	513 (2.8)	0 (0.0)	1042 (3.1)
Substance abuse disorders	6 (2.7)	1839 (10.1)	31 (17.7)	2374 (7.0)
Depression/anxiety	32 (14.5)	2920 (16.1)	79 (45.1)	8379 (24.6)
Concomitant medication use, n (%)				
Diabetes therapies	3 (1.4)	1154 (6.4)	3 (1.7)	1335 (3.9)
Psychiatric/neurologic therapies	30 (13.6)	2961 (16.3)	64 (36.6)	7641 (22.4)
Steroids	4 (1.8)	738 (4.1)	0 (0.0)	555 (1.6)
Hormone therapy/contraception	0 (0.0)	581 (3.2)	2 (1.1)	1345 (3.9)
Appetite stimulants/suppressants	14 (6.4)	704 (3.9)	35 (20.0)	2421 (7.1)
Antihypertensives	5 (2.3)	2345 (12.9)	12 (6.9)	3624 (10.6)
Statins	1 (0.5)	1548 (8.5)	1 (0.6)	2478 (7.3)

Abbreviations: CDHP/HDHP, consumer-directed health plan/high-deductible health plan; EPO, exclusive provider organization; HMO, health maintenance organization; POS, point of service; PPO, preferred provider organization; PrEP, pre-exposure prophylaxis; QCI, Quan-Charlson Comorbidity Index.

^a^Includes comprehensive/indemnity, EPO, missing or unknown.

^b^Data were available only from January to April 2021.

**Figure 3.**
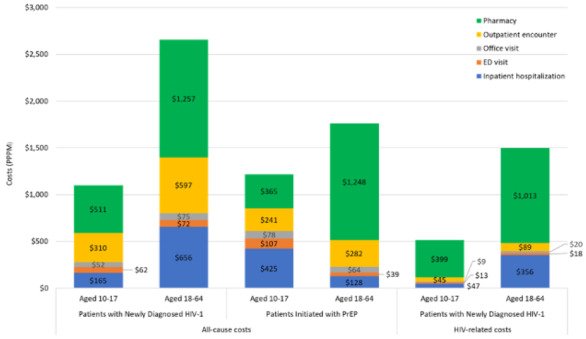
All-cause and HIV-Related Costs Abbreviations: ED, emergency department; PrEP, pre-exposure prophylaxis.

Newly diagnosed adult HIV-1 patients were followed for a mean (SD) of 17.8 (15.1) months. Mean number (SD) of all-cause inpatient hospitalizations, ED visits, office visits, other outpatient encounters (including laboratory testing), and pharmacy prescriptions were 0.02 (0.09), 0.04 (0.08), 0.62 (0.60), 4.37 (6.18), and 1.71 (1.91) PPPM, respectively, while the number of HIV-related encounters was lower, ranging from 0.01 (0.06) inpatient hospitalizations and 0.01 (0.04) ED visits to 1.29 (2.26) other outpatient encounters PPPM **(Table 2).** Mean (SD) total all-cause costs were $2657 ($5954) PPPM, of which 47% was driven by all-cause pharmacy costs of $1257 ($1845) PPPM. Total HIV-related pharmacy costs constituted 67% of total HIV-related costs **(Table 3; Figure 3**); however, only 37% of the cohort had at least 1 HIV-related pharmacy prescription during follow-up **(Table 2)**.

**Table 2.** Healthcare Resource Utilization

	**Age Group**			
	**Patients With Newly Diagnosed HIV-⁠1**		**Patients Initiated With PrEP**	
	**10-17 y**	**18-64 y**	**10-17 y**	**18-64 y**
Total No. of patients	220	18 154	175	34 123
Length of follow-⁠up^a^ (mo), mean (SD)	20.9 (15.7)	17.8 (15.1)	18.4 (14.6)	16.8 (14.1)
All-cause healthcare resource utilization				
Patients with ≥1 admission/visit, n (%)				
Inpatient hospitalization	27 (12.3)	4106 (22.6)	44 (25.1)	2324 (6.8)
ED visit	74 (33.6)	5386 (29.7)	73 (41.7)	6072 (17.8)
Office visit	206 (93.6)	17 028 (93.8)	156 (89.1)	29 413 (86.2)
Outpatient encounter^b^	215 (97.7)	17 707 (97.5)	157 (89.7)	30 331 (88.9)
Pharmacy prescriptions	173 (78.6)	16 030 (88.3)	175 (100.0)	34 118 (100.0)
No. of admissions/visits PPPM, mean (SD)				
Inpatient hospitalization	0.01 (0.06)	0.02 (0.09)	0.03 (0.07)	0.01 (0.05)
ED visit	0.04 (0.07)	0.04 (0.08)	0.07 (0.10)	0.02 (0.06)
Office visit	0.40 (0.35)	0.62 (0.60)	0.60 (0.57)	0.52 (0.49)
Outpatient encounter^b^	2.29 (2.61)	4.37 (6.18)	2.53 (2.12)	3.09 (3.12)
Pharmacy prescriptions	0.65 (0.90)	1.71 (1.91)	1.47 (1.50)	1.68 (1.57)
HIV-related healthcare resource utilization				
Patients with ≥1 admission/visit, n (%)				
Inpatient hospitalization	11 (5.0)	1774 (9.8)	NA	NA
ED visit	21 (9.5)	1855 (10.2)	NA	NA
Office visit	75 (34.1)	9159 (50.5)	NA	NA
Outpatient encounter^b^	185 (84.1)	15 817 (87.1)	NA	NA
Pharmacy prescriptions	34 (15.5)	6708 (37.0)	NA	NA
No. of admissions/visits PPPM, mean (SD)				
Inpatient hospitalization	0.004 (0.02)	0.01 (0.06)	NA	NA
ED visit	0.01 (0.03)	0.01 (0.04)	NA	NA
Office visit	0.06 (0.15)	0.15 (0.26)	NA	NA
Outpatient encounter^b^	0.65 (1.32)	1.29 (2.26)	NA	NA
Pharmacy prescriptions	0.14 (0.38)	0.34 (0.51)	NA	NA

Abbreviations: ED, emergency department; NA, not applicable; PPPM, per patient per month; PrEP, pre-exposure prophylaxis.

^a^Newly diagnosed HIV-1 patients were followed from the diagnosis index date to the earlier of end of continuous enrollment in the database or end of study period. Patients initiated with PrEP were followed from the treatment index date to the earliest of HIV-1 diagnosis, end of continuous enrollment, or end of the study period.

^b^Includes laboratory testing.

**Table 3.** Healthcare Costs

	**Age Group**							
	**Patients With Newly Diagnosed HIV-1**				**Patients Initiated With PrEP**			
	**10-⁠17 y (n = 220)**		**18-⁠64 y (n = 18 154)**		**10-⁠17 y (n = 175)**		**18-⁠64 y (n = 34 123)**	
	**Mean (SD)**	**Median (IQR)**	**Mean (SD)**	**Median (IQR)**	**Mean (SD)**	**Median (IQR)**	**Mean (SD)**	**Median (IQR)**
All-cause costs PPPM								
Total costs	$1101 (2147)	$225 (73, 915)	$2657 (5954)	$1383 (292, 3441)	$1217 (1501)	$755 (399, 1224)	$1761 (1938)	$1625 (745, 2167)
Total medical costs	$589 (1641)	$157 (61, 390)	$1399 (5497)	$343 (143, 971)	$852 (1391)	$437 (131, 807)	$513 (1556)	$195 (87, 456)
Inpatient hospitalization	$165 (764)	$0 (0, 0)	$656 (4551)	$0 (0, 59)	$425 (1098)	$0 (0, 288)	$128 (1123)	$0 (0, 0)
ED visit	$62 (130)	$0 (0, 57)	$72 (246)	$0 (0, 63)	$107 (172)	$31 (0, 152)	$39 (157)	$0 (0, 7)
Office visit	$52 (68)	$36 (19, 60)	$75 (91)	$53 (28, 92)	$78 (98)	$52 (30, 98)	$64 (69)	$47 (25, 82)
Outpatient encounter	$310 (1181)	$61 (22, 181)	$597 (2261)	$162 (66, 425)	$241 (336)	$129 (44, 309)	$282 (733)	$105 (41, 273)
Total pharmacy costs	$511 (1181)	$17 (1, 145)	$1257 (1845)	$203 (13, 2704)	$365 (460)	$365 (117, 433)	$1248 (1052)	$1259 (426, 1821)
HIV-related costs PPPM								
Total costs	$512 (1217)	$6 (2, 38)	$1497 (4463)	$43 (5, 2874)				
Total medical costs	$114 (358)	$6 (2, 38)	$484 (4111)	$29 (4, 159)				
Inpatient hospitalization	$47 (277)	$0 (0, 0)	$356 (4020)	$0 (0, 0)				
ED visit	$13 (72)	$0 (0, 0)	$18 (141)	$0 (0, 0)				
Office visit	$9 (22)	$0 (0, 5)	$20 (41)	$2 (0, 29)				
Outpatient encounter	$45 (143)	$3 (0, 16)	$89 (387)	$15 (2, 75)				
Total pharmacy costs	$399 (988)	$0 (0, 0)	$1013 (1410)	$0 (0, 2513)				

Abbreviations: ED, emergency department; IQR, interquartile range; PPPM, per patient per month; PrEP, pre-exposure prophylaxis.

All dollar estimates were inflated to 2020 US dollars using the Medical Care Component of the Consumer Price Index.

### Initiated With PrEP: Adults

Of adults initiated with PrEP, the mean (SD) age was 34.7 (11.0) years; 91.0% were male. Most patients were from the South (41.0%), followed by the Northeast (21.9%) and West (20.5%). The most common commercial plan type was PPO (48.0%) **(Table 1).** During the baseline period, mean (SD) QCI score was 0.2 (0.5), and comorbidities that were reported in more than 10% of the cohort included depression/anxiety, hyperlipidemia, and hypertension **(Table 1).**

Adult patients initiated with PrEP were followed for a mean (SD) of 16.8 (14.1) months. All-cause inpatient hospitalizations, ED visits, office visits, other outpatient encounters (including laboratory testing), and pharmacy prescriptions occurred at a mean rate (SD) of 0.01 (0.05), 0.02 (0.06), 0.52 (0.49), 3.09 (3.12), and 1.68 (1.57) PPPM, respectively **(Table 2).** Mean (SD) total all-cause costs were $1761 ($1938) PPPM, of which 71% was driven by all-cause pharmacy costs of $1248 ($1052) PPPM **(Table 3).**

### Exploratory Analysis of Adolescent Patients

Trends in the baseline demographics of adolescents with newly diagnosed HIV-1 were generally consistent with those of adults **(Table 1)**. In contrast, in patients initiated with PrEP, the majority (91.0%) of adults were male, while only 30.3% of adolescents were male. Baseline comorbidities of note in adolescents with newly diagnosed with HIV-1 and in those initiated with PrEP included depression/anxiety (14.5% and 45.1%, respectively) and substance abuse disorders (2.7% and 17.7%, respectively), and the use of psychiatric/neurologic therapies was observed in 13.6% and 36.6% of adolescents, respectively **(Table 1)**.

Adolescents with newly diagnosed HIV-1 and those initiated with PrEP were followed for a mean (SD) of 20.9 (15.7) months and 18.4 (14.6) months, respectively. During follow-up, mean (SD) total all-cause costs were $1101 ($2147) PPPM for adolescents newly diagnosed with HIV-1, with total pharmacy costs accounting for 46.4%; however, 78.6% of patients had at least 1 all-cause pharmacy prescription, but only 15.5% had an HIV-related pharmacy prescription. In adolescents initiated with PrEP, mean (SD) pharmacy costs ($365 [$460]) accounted for 30.0% of total all-cause costs ($1217 [$1501]); 100% of this cohort had at least 1 all-cause pharmacy prescription, due to the cohort identification criteria **(Tables 2 and 3).**

## DISCUSSION

By leveraging claims data, this study described the demographics and clinical characteristics of patients with newly diagnosed HIV-1 and those initiating PrEP for HIV prevention and provided a comprehensive overview of the economic burden of disease in the study patients. By including multiple cohorts in our study, we aimed to uncover potential trends in characteristics and economic outcomes among PLWH and those at risk of being infected by HIV-1 in a commercially insured population.

First, of PLWH, our study included those who were newly diagnosed during the study period and showed that these patients are incurring costs early in their course of disease. While PLWH with advanced disease and an extensive treatment history have been shown to have higher all-cause and HIV-related costs and HRU,^23^ the mean total all-cause costs among patients with newly diagnosed HIV-1 were $2657 PPPM during our study period, which is still similar to costs due to other chronic conditions, as evidenced by a study in patients with heart disease that did not differentiate between recent and long-term diagnoses.^24^

Next, our study calls attention to important considerations for the use of ART for HIV treatment. In this study, only 37.0% of newly diagnosed adults and 15.5% of newly diagnosed adolescents had at least 1 HIV-related prescription during follow-up; this may indicate undertreatment or delayed treatment unless patients obtained medication through channels other than their commercial insurance. Further research would be needed to validate this, particularly given the emphasis on rapid ART initiation in current guidelines for HIV care,^25^ and 2019 US estimates that approximately 81% of people are linked to HIV medical care within 1 month of diagnosis.^1^

With regard to those receiving PrEP, our study of claims data was able to assess characteristics of interest in new PrEP users that may be useful in informing targeted efforts to increase PrEP use in certain at-risk patient groups. For example, there was a noticeable difference between adults and adolescents initiated with PrEP, in terms of gender: adults who initiated PrEP were predominantly male (91.0%), while 69.7% of adolescents who initiated PrEP were female. In comparison, the CDC also reports greater PrEP coverage for males vs females aged 16 years or older, but data are not available for younger people.^1^ While our results are of interest in addressing this gap, the sample sizes for adolescents with newly diagnosed HIV-1 and those initiated on PrEP were very small, and the exploratory results for those cohorts should be interpreted with caution.

In addition to age and gender, other disparities have been identified in the use of HIV PrEP, with lower use among racial/ethnic minorities and patients living in the South.^26,27^ Recent research indicates that PrEP use may be low in part due to stigma, lack of awareness, and concerns related to cost.^28,29^ It would be beneficial to conduct further research to identify reasons for specific disparities across demographic and socioeconomic groups, given the need to improve PrEP coverage overall. Although a previous study found an increasing trend in PrEP utilization between 2010 and 2014,^18^ the CDC estimates^1^ that HIV PrEP is still underused.

It has been established that efficacy is highly dependent on medication adherence.^26,28^ While assessment of medication adherence was beyond the scope of the current study, poor adherence to PrEP may contribute to future HIV infection, antiretroviral drug resistance, and potentially higher HRU and costs than those reported here. Due to timing of the current study, patients receiving long-acting injectable cabotegravir were not included, and, thus, potential impact of novel PrEP delivery methods is not reflected here. Convenient and affordable means of HIV prevention remain an important unmet need in the United States, but future research on trends in PrEP use following availability of long-acting formulations will be needed.

Certain clinical characteristics, which may also play a role in HRU and costs in these populations, were evaluated in our analysis of claims data. In our study, 16.1% of adults with newly diagnosed HIV-1 and 24.6% of adults initiated with PrEP were reported to have depression/anxiety during the baseline period. Although the sample size was small in the exploratory analyses of adolescents, 14.5% and 45.1% of those with newly diagnosed HIV-1 and those initiated with PrEP, respectively, had medical claims with diagnoses of depression/anxiety. Depression occurs frequently among PLWH; one meta-analysis reported occurrence of depressive symptoms ranging from 13% to 78% across studies, with a pooled rate of 39%.^30^ These estimates are greater than those found in our study, suggesting that certain diagnoses, including depression and anxiety, may be underrepresented in claims data and the actual degree of impact may be greater than that reported in our study.

This study included a considerable sample size of patients from a large administrative claims database, which is representative of the employer-sponsored, commercially insured population in the United States. However, certain limitations should be considered when interpreting these findings. As with all retrospective claims database analyses, administrative claims data were collected for the purpose of facilitating payment for healthcare services, and definitive diagnoses are not available in the administrative claims database. While our methodology was carefully designed to identify the newly diagnosed PLWH using diagnosis codes associated with medical claims, there is potential for misclassification or miscoding in any study using real-world data. Vital sign values are not available in the data; for example, obesity was identified in this study using diagnosis codes rather than recorded BMI values, and the condition is likely underreported. Laboratory test results are also not available in the data; therefore, factors such as viral load, which may impact overall disease severity, HRU, and cost burden, were not assessed. Cash payments for prescriptions and over-the-counter products are not captured in the claims data, nor are medications obtained through patient assistance programs or otherwise not processed through insurance. Costs represented in the claims data reflect the paid amounts of adjudicated claims to individual hospitals and providers. Indirect costs or potential caregiver burden was not considered due to the unavailability of this information in the dataset.

The proportion of patients reported to be in a particular geographic region may be influenced by overall representation of geographic regions in the dataset, as other studies have found that the largest proportion of patients in this dataset were in the South.^31,32^ This study was composed of patients covered by commercial insurance; therefore, the results may not be generalizable to patients with other forms of insurance or those without health insurance coverage. The findings of this study, which focused on incident HIV patients and PrEP users, may not be generalizable to the corresponding prevalent patient groups, and HRU and costs may differ for patients with advanced disease with or without viral suppression or with greater duration of infection prophylaxis.

We did not measure medication adherence in this study, which may further impact outcomes in this population. To identify the cohort of patients with newly diagnosed HIV-1 in this study, we excluded those with prior use of ART. Thus, it is possible that high-risk patients previously receiving PrEP or postexposure prophylaxis were excluded from the cohort even if they went on to become HIV-positive. Previous PrEP or postexposure prophylaxis use, as well as disease severity, could be confounders for future research including predictive models of HIV infection and risk comparisons.

Finally, this study did not address other social, economic, and public health factors, particularly the global COVID-19 pandemic, which occurred during the study period and could have potentially impacted HRU and costs, occurrence of comorbidities, or other variables assessed here.

## CONCLUSIONS

This study provided data on economic burden in newly diagnosed HIV-1 and newly initiated PrEP populations. Despite ART availability and advances in those treatments, PLWH continue to incur considerable healthcare resources and costs. In addition, this study found that a low proportion of newly diagnosed PLWH had filled prescriptions for ART. Existing literature shows that HIV PrEP is underutilized in those at risk of infection. While our study assessed a modest follow-up period in new patients, it suggests that there is still a need to identify and implement interventions to reduce burden in these populations. Specifically, this warrants an increase in appropriate and effective use of ART for both HIV treatment and PrEP, development of alternative preventions strategies such as vaccines, further work toward improved management of associated comorbidities, and removal of barriers such as provider awareness and patient adherence to the available treatment options.

### Author Contributions

C.C., P.D., A.C., and B.T. were involved in study design, analysis, and interpretation. C.C. was responsible for the statistical analyses.

### Disclosures

C.C., P.D., and A.C. are employees of Janssen Scientific Affairs, LLC (a Johnson & Johnson company) and hold stock in Johnson & Johnson. B.T. has served as a paid consultant to ViiV Healthcare, GSK, Gilead, Merck, and Janssen Scientific Affairs, LLC.

## Supplementary Material

Supplementary Online Material
