# Supplementary material for: Economic Burden of HIV in a Commercially Insured Population in the United States
Source: J Health Econ Outcomes Res. 2023 Jan 19;10(1):10–9. doi: 10.36469/001c.56928 (PMC9865714; doi:10.36469/001c.56928)
Supplement: Supplementary Online Material [file jheor_2023_10_1_56928_135487.pdf]

Journal of Health Economics and Outcomes Research

Online Supplementary Material

Economic Burden of HIV in a Commercially Insured Population in the United States. *JHEOR*. **2023;10(1):10-19.** doi:10.36469/jheor.2023.56928

Table S1: Codes for Patient Identification

Table S2: Codes for Antiretroviral Therapy

Table S3: Codes for Hepatitis B Medications

Table S4: Codes for Comorbid Conditions

Table S5: Codes for Concomitant Medications

This supplementary material has been provided by the authors to give readers additional information about their work.

This is an open-access article distributed under the terms of the Creative Commons Attribution 4.0 International License (CCBY-4.0). View this license's legal deed at http://creativecommons.org/licenses/by/4.0 and legal code at http://creativecommons.org/licenses/by/4.0/legalcode for more information.

Table S1. Codes for Patient Identification

Diagnosis	ICD-9-CM Codes	ICD-10-CM Codes
HIV-1	042, 795.71, V08	B20, R75, Z21
HIV-2	079.53	B97.35
Hepatitis B	070.20-070.23, 070.30-070.33, 070.42, 070.52	B16.0-B16.9, B17.0, B18.0, B18.1, B19.10, B19.11
Medication	GPI Codes	
Emtricitabine/tenofovir disoproxil fumarate	1210990230%	
Emtricitabine/tenofovir alafenamide	12109902290320	

Abbreviations: GPI, generic product identifier; ICD-9-CM, *International Classification of Diseases, Ninth Revision, Clinical Modification*; ICD-10-CM, *International Classification of Diseases, Tenth Revision, Clinical Modification.*

Table S2. Codes for Antiretroviral Therapy

ARV Class	ARV Subclass	Generic Name	GPI Codes	HCPCS Codes
PI	PI	ATV	12104515%	
	PI	DRV	12104520%	
	PI	FPV	12104525%	
	PI	SQV	12104580%	S0140
	PI	TPV	12104585%	
	PI	Amprenavir	12104510%	
	PI	IDV	12104530%	
	PI	NFV	12104545%	
	PE(PI)	Ritonavir	12104560%	
	PI/PE	ATV/COBI	12109902220330	
	PI/PE	DRV/COBI	12109902270320	
	PI/PI	LPV/r	1210990255%	
	PI/PE/NRTI/TAF	DRV/COBI/FTC/TAF	12109904200320	
INI	INI	DTG	12103015%	
	INI	RAL	12103060%	
	INI	EVG	1210302000%	
	INI/NRTI/NRTI	DTG/ABC/3TC	12109903150320	
	INI/NNRTI	DTG/RPV	12109902280320	
	INI/PE/NRTI/NRTI	EVG/COBI/FTC/TDF	1210990430%	
	INI/NRTI/TAF	BIC/FTC/ TAF	12109903240330	
	INI/PE/NRTI/TAF	EVG/COBI/FTC/TAF	1210990429%	
	INI/NRTI	DTG/3TC	12109902260320	
NRTI	TAF	TAF	12352083200320	
	NRTI	ABC	12105005%	
	NRTI	FTC	12106030%	
	NRTI	3TC	12106060%	
	NRTI	TDF	12108570%	
	NRTI	ZDV	12108085%	J3485, S0104
	NRTI	ddC	12106085%	
	NRTI	d4T	12108070%	
	NRTI	ddI	12105015%	S0137
	NRTI/TAF	FTC/TAF	12109902290320	
	NRTI/NRTI	ABC/3TC	1210990220%	
	NRTI/NRTI/NRTI	ABC/3TC/ZDV	1210990320%	

Table S2. Codes for Antiretroviral Therapy, *cont'd*

ARV Class	ARV Subclass	Generic Name	GPI Codes	HCPCS Codes
	NRTI/NRTI	FTC/TDF	1210990230%	
	NRTI/NRTI	3TC/TDF	12109902470330	
	NRTI/NRTI	3TC/TDF	N/A	
	NRTI/NRTI	3TC/ZDV	1210990250%	
NNRTIs	NNRTI	DOR	12109025000320	
	NNRTI	EFV	12109030%	
	NNRTI	ETR	12109035%	
	NNRTI	NVP	12109050%	
	NNRTI	RPV	12109080100320	
	NNRTI	DLV	12109020%	
	NNRTI/NRTI/NRTI	EFV/FTC/TDF	1210990330%	
	NNRTI/NRTI/NRTI	EFV/3TC/TDF	12109903330340	
	NRTI/NNRTI/NRTI	FTC/RPV/TDF	1210990340%	
	NNRTI/NRTI/NRTI	DOR/3TC/TDF	12109903270320	
	NRTI/NNRTI/TAF	FTC/RPV/TAF	12109903390320	
Others	Fusion inhibitor	T20	12102530%	J1324
	CCR5 antagonist	MVC	12102060%	
	PE	COBI	12109530000320	
	Post-attachment inhibitors	IBA	12102240302020	

Source: HIV.gov. *Guidelines for the Use of Antiretroviral Agents in Adults and Adolescents with HIV.* Updated September 21, 2022. https://clinicalinfo.hiv.gov/en/guidelines/adult-and-adolescent-arv/whats-new-guidelines

Abbreviations: 3TC, lamivudine; ABC, abacavir; ARV, antiretroviral; ATV, atazanavir; BIC, bictegravir; COBI, cobicistat; d4T, stavudine; ddC, zalcitabine; ddI, didanosine; DRV, darunavir; DLV, delavirdine; DOR, doravirine; DTG, dolutegravir; EFV, efavirenz; ETR, etravirine; EVG, elvitegravir; FPV, fosamprenavir; FTC, emtricitabine; GPI, generic product identifier; HCPCS, healthcare common procedure coding system; IBA, ibalizumab; IDV, indinavir; INI, integrase inhibitor; LPV/r, lopinavir/ritonavir; MTR, multi-tablet regimen; MVC, maraviroc; NFV, nelfinavir; NNRTI, non-nucleoside reverse transcriptase inhibitor; NRTI, nucleoside/nucleotide reverse transcriptase inhibitor; NVP, nevirapine; PE, pharmacokinetic enhancer; PI, protease inhibitor; RAL, raltegravir; RPV, rilpivirine; SQV, saquinavir; STR, single-tablet regimen; T20, enfuvirtide; TAF, tenofovir alafenamide; TDF, tenofovir disoproxil fumarate; TPV, tipranavir; ZDV, zidovudine.

Table S3. Codes for Hepatitis B Medications

Medication	GPI	HCPCS
Tenofovir disoproxil	1210857010%	
Tenofovir alafenamide	1235208320%	
Entecavir	12352030%	
Telbivudine	12352080%	
Adefovir dipivoxil	1235201510%	
Lamivudine	12106060%	
Interferon alpha	2170006010%, 2170006020%, 2170006030%	J9213, J9214, J9215, S0145, S0146, S0148

Source: Hepatitis B Foundation. Approved drugs for adults. https://www.hepb.org/treatment-and-management/treatment/approved-drugs-for-adults/

Abbreviations: GPI, generic product identifier; HCPCS, healthcare common procedure coding system.

Table S4. Codes for Comorbid Conditions

Condition	ICD-9-CM Codes	ICD-10-CM Codes
Prediabetes	790.29	R73.03
Type 2 diabetes	250.% (fifth digit with 0 or 2 only)	E11.%
Myocardial infarction	410.%, 412.%	I21.%, I22.%, I25.2%
Peripheral vascular disease	440.2%, 440.3%, 440.4%, 440.8%, 440.9%, 443.22, 443.81, 443.89, 443.9, 444.22, 444.81, 445.02	E08.5%, E10.5%, E11.5%, E13.5%, I70.2%, I70.3%, I70.4%, I70.5%, I70.6%, I70.7%, I70.8%, I70.9%, I73.89, I73.9%, I79.8%, I74.3%, I74.5%, I75.029
Congestive heart failure	402.01, 402.11, 402.91, 404.91, 404.93, 391.8%, 398.91, 392.0%, 428.%	I01.8%, I09.81, I02.0%, I50.%
Hypertension	401.% - 405.%	I10.% - I16.%
Hyperlipidemia	272.0%, 272.1%, 272.2%, 272.3%, 272.4%	E78.0%, E78.1%, E78.2%, E78.3%, E78.4%, E78.5%
Obesity	278.00, 278.01, 278.03, V85.3%, V85.4%	E66.0%-E66.2%, E66.8%, E66.9%, Z68.3%, Z68.4%
NASH/nonalcoholic fatty liver	571.8% (use this only before 10/1/2015)	K75.81 (NASH), K76.0 (non-alcoholic fatty liver)
Cancer	140.% - 172.%, 174.% - 195.8%, 200.% - 208.%	C00.%-C26.%, C30.%-C34.%, C37.%-C41.%, C43.%, C45.%-C58.%, C60.%-C76.%, C81.%-C85.%, C88.%, C90.%
Lipodystrophy	272.6	E88.1%
Opportunistic infections, including pneumocystis carinii pneumonia, Kaposi's sarcoma, cryptosporidiosis, herpes simplex, cryptococcosis, toxoplasmosis	136.3%, 176.%, 007.4%, 054%, 117.5%, 130%	B59%, C46%, A07.2%, B00%, B45%, B58%
Depression and anxiety	300.0%, 296.2%, 296.3%, 300.4%, 311.%	F41.%, F32.%, F33.%, F32.%, F33.%
Substance abuse disorders	304.%, 305.%	F10.% - F19.%

Abbreviations: ICD-9-CM, *International Classification of Diseases, Ninth Revision*; ICD-10-CM, *International Classification of Diseases, Tenth Revision*; NASH, nonalcoholic steatohepatitis.

Table S5. Codes for Concomitant Medications

Medication	GPI Codes	HCPCS Codes
Diabetes therapies		
Insulin	2710%, 279910%	J1815, J1817, E0784, S5550-S5571, G9147, S9353
Thiazolidinediones	2760%, 279940%, 279978%, 279980%	
Sulfonylureas	2720%, 279970%, 279978%	
Biguanides/meglitinides	2725%, 279925%, 279960%, 279970%, 279980%, 279988%, 279990%, 2728%, 279950%	
DPP-4 inhibitors	2755%, 279925%, 279930%, 279940%, 279965%	
GLP-1 receptor agonists	2717%, 279910%	
α-Glucosidase inhibitors	2750%	
Psychiatric/neurologic therapies		
Tricyclic antidepressants	5820%	
Selective serotonin reuptake inhibitors	5816%	
Antipsychotics	5907%, 5910%, 5915%, 5916%, 5920%, 5925%, 5930%, 5940%, 5950%	
Antiseizure/anticonvulsants (valproic acid, carbamazepine, gabapentin, topiramate, zonisamide, lamotrigine)	72500010%, 72500020%, 72500030%, 72600020%, 72600030%, 72600075%, 72600090%, 72600040%	
Others (bupropion, nefazodone, lithium, mirtazapine)	58300040%, 58120050%, 59500010%, 58030050%	
Steroids	22%, 8915%	J0702, J7624, J7622, J7633, J7627, J7634, J7626, J8540, J1094, J1100, J7312, J7637, J7638, J1700, J1710, J1720, J1020, J1030, J1040, J2920, J2930, J7509, J2650, J7510, J7506, J7512, J3300, J3301, J3302, J3303, J7683, J7684
Hormone therapy/contraception (estrogen, testosterone, progesterone, tesamorelin)	2400%, 2499%, 231000%, 260000%, 30150085%	
Appetite stimulants/suppressants (phentermine, topiramate, methylphenidate, amphetamine/ dextroamphetamine, megestrol, oxandrolone, dronabinol)	61200070%, 72600075%, 61400020%, 61109902%, 21404020%, 23200040%, 50300030%	
Statins	3940%	
Antihypertensives	36%	J0210, J2760, J0360, S0139, J1730, J2670

Abbreviations: GPI, generic product identifier; HCPCS, healthcare common procedure coding system.
